# Gnathic Osteosarcoma: Clinical, Radiologic, and Pathologic Review of Bone Beard Tumor

**DOI:** 10.1200/JGO.2016.006494

**Published:** 2016-08-31

**Authors:** Abhishek Mahajan, Richa Vaish, Subhash Desai, Supreeta Arya, Nilesh Sable, Anil K. D’cruz

**Affiliations:** **All authors:** Tata Memorial Hospital, Tata Memorial Centre, Mumbai, Maharashtra, India.

## INTRODUCTION

Gnathic (mandibular) osteosarcomas (OSs) account for approximately 6.5% of all OS, and most of them are secondary in nature, occurring in patients with Paget disease or fibrous dysplasia or as a late sequela to craniofacial radiotherapy.^1,2^ Primary OS of the jaw is rare and presents approximately two decades later than its appendicular counterpart; metastasis is uncommon with primary OS, and it has a better prognosis than appendicular OS. Radiologic depiction of a sunburst pattern of new bone formation is characteristic.^1,2^ Early diagnosis and complete tumor resection are mandatory to improve the prognosis of jaw OS.

## CASE REPORT

**Fig 1:**
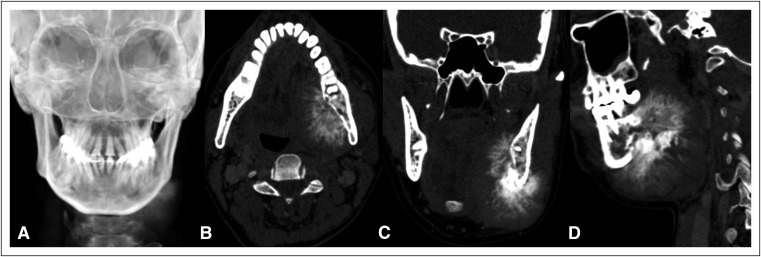
(A) Reconstructed radiograph and (B, C, and D) computed tomography images (B: axial; C: coronal; and D: sagittal in bone algorithm) reveal a minimally expansile lytic lesion of the left hemimandible involving its angle and body with erosions of the medial cortex and new bone formation perpendicular to the mass forming the typical sunburst pattern.

A 56-year-old woman presented with left side cheek swelling of 1.5 months in duration. On clinical examination, expansile hard swelling of left hemimandible was noted that was seen to be both intraoral as well as in the neck. A reconstructed radiograph (Fig 1A) showed a bone-forming lesion along the left side of the mandible. Computed tomography (CT) imaging of the face and neck was performed for further characterization. CT imaging (Figs 1B, 1C, and 1D) revealed a minimally expansile lytic lesion of the left hemimandible involving its angle and body with erosions of the medial cortex and new bone formation perpendicular to the mass forming the typical sunburst pattern. The patient also complained of a lump in the left breast for which bilateral mammograms were performed that revealed a Breast Imaging Reporting and Data System category 4 lesion in the left retroareaolar region. A biopsy of the left breast mass was done, and histopathology showed infiltrating duct carcinoma (grade 2). On the basis of the imaging findings of the mandibular lesion, diagnosis of aggressive bone lesion was made with the possibility of metastasis from breast primary. Primary OS was considered as the differential diagnosis. Needle biopsy of the mandibular swelling was performed, and the pathologic diagnosis was OS. The patient was planned for excision of both the left mandibular lesion and the left breast mass; however, the patient defaulted the treatment.

**Fig 2:**
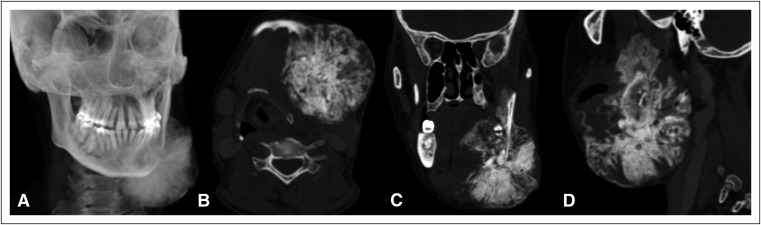
(A) Reconstructed radiograph and (B, C, and D) computed tomography images (B: axial; C: coronal; and D: sagittal in bone algorithm) reveal significant increases in the extent and size of the mass, particularly the osseous periosteal reaction.

**Fig 3:**
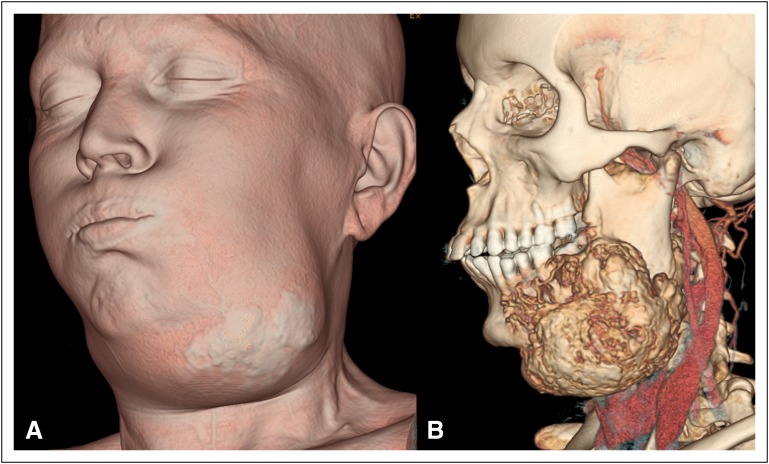
(A and B) Three-dimensional volume-rendered computed tomography images showing the bone beard tumor along the mandible.

**Fig 4:**
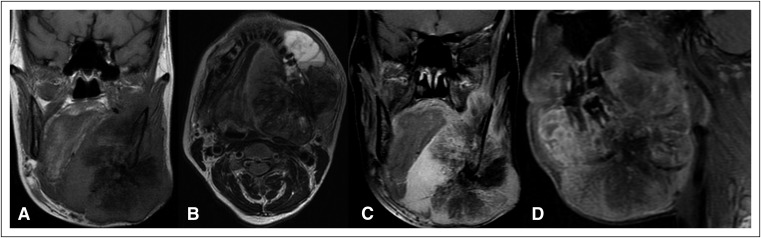
(A) Plain T1-weighted coronal, (B) T2-weighted axial, and (C) postcontrast T1-weighted magnetic resonance images of the neck reveal an expansile lesion in the left hemimandible extending into the submandibular region, floor of mouth, parapharyngeal space, and masticator space. T1- and T2-weighted images show large areas of low intensity with intervening enhancing soft tissue component within the mass giving a sunburst appearance.

Four months later, the patient presented with rapid progression of the left mandibular mass. A repeat reconstructed radiograph (Fig 2A) and CT imaging (Figs 2B, 2C, and 2D) revealed a significant increase in the extent and size of the mass, particularly the osseous periosteal reaction (Figs 3A and 3B show three-dimensional volume-rendered images of the bone beard tumor). Magnetic resonance imaging (MRI; Fig 4) of the neck was performed for planning the extent of resection, which revealed an expansile lesion in the left hemimandible extending into the submandibular region, floor of mouth, oral cavity, parapharyngeal space, and masticator space. T1- and T2-weighted images revealed large areas of low intensity with an intervening enhancing soft tissue component within the mass giving a sunburst appearance representing new bone formation, which was suggestive of an aggressive type of periosteal reaction.

The patient underwent left hemimandibulectomy with free fibula osteocutaneous flap reconstruction. The histopathology revealed OS with approximately 75% tumor necrosis. The left breast mass was excised and subjected to histopathology examination, which revealed infiltrating duct carcinoma with predominant ductal carcinoma in situ. Eight months after surgery, the patient presented with diffuse skeletal pain and dyspnea. Chest radiograph revealed pleural effusion, and technetium-99m–methylene diphosphonate bone scan showed multiple-site skeletal metastases with soft tissue tracer uptake in the left-sided pleural space with effusion suggestive of pleural involvement. Oral and neck examination did not show any evidence of recurrent or residual disease. The patient was managed for metastatic disease from the breast primary tumor.

## DISCUSSION

OS of the jaw is rare and composes approximately 6.5% of all OSs and 1% of all malignant tumors of the head and neck. These tumors present later than extremity OS, are characterized by a higher propensity for local recurrence and absence of distant and pulmonary metastases, and are refractive to chemotherapy.^1^ Two variants have been described in the literature, primary and secondary, of which secondary mandibular OS is more common. Secondary mandibular OS usually occurs in association with Paget disease, fibrous dysplasia, or Ollier disease or as late sequela to craniofacial radiotherapy. The exact cause of OS is unknown; however, a number of risk factors have been described. Rapid bone growth has been enumerated to be one of the predisposing factors in the etiopathogenesis of OS.^3,4^ The secondary variant suggests an association between OS in general and excessive bone cellular activity. The reported incidence of OS is significantly higher in patients with retinoblastoma, which suggests the role of the *RB1* gene in tumorigenesis of both diseases. Other syndromic associations include Li-Fraumeni syndrome, Bloom syndrome, Werner syndrome, Rothmund-Thomson syndrome type 2, Diamond-Blackfan anemia, and RAPADILINO (radial ray defect, patellae hypoplasia or aplasia and cleft or highly arched palate, diarrhea and dislocated joints, little size and limb malformations, and long slender nose and normal intelligence) syndrome.^2,3^

Males are more commonly affected than females. Swelling, loss and displacement of local teeth, spasm, and local parasthesia are common presenting complaints and are often misdiagnosed as chronic pulpitis or chronic periodontitis. The condition is usually not associated with pain. Most patients present after dental treatment and mostly relate the symptoms to previous tooth extraction.^3,4^ The radiographic appearances are varied, ranging from osteolytic pattern to mixed osteogenic pattern. The osteogenic pattern almost always shows an area of the typical sunburst appearance, which on radiography is seen as stippled bone pattern with destruction of the cortical outlines and perpendicular striae (Sharpey’s fiber) of periosteal reaction. This pattern suggests the rapid tumoral growth that gives limited time for periosteum to lay down a new layer of protective bone. The common mimics of this radiologic pattern include odontomas, odontogenic myomas, solitary plasmacytomas, squamous cell carcinoma of the mandible, central hemangioma, fibrous dysplasia, and metastatic lesions from primary colonic malignancies. Other nonspecific features include widening of the periodontal ligament space and attenuation of the lamina dura.^5,6^ Valuable adjuncts for not only evaluating the extent of the tumor but also assessing its relationship with neighboring tissues include CT, as a result of its superior bone architectural detection, and MRI, as a result of its ability to image the soft tissues. On CT imaging, the mass shows thin irregular spicules of new bone formation that are perpendicular to the epicenter of the lesion, giving the classic sunburst appearance. Similar appearance can be appreciated on MRI as well, where the ossified newly formed periosteal bone reaction shows low signal on all of the sequences and the actively proliferating soft tissue mass is seen as enhancing intervening areas.^5-7^ Three-dimensional volume-rendered CT reconstruction and MRI play an important role in planning the surgical management.

The local aggressiveness of mandibular OS is greater than that of appendicular OS despite the same histology. The common histologic variants, on the basis of the type of extracellular matrix produced by the tumoral cells, are the same as those affecting the appendicular skeleton and include osteoblastic, fibroblastic, chondroblastic, and mixed OS. Other less common histologic variants are myxomatous, telangiectatic, epithelioid, fibrous histiocytoma–like, giant-cell, small-cell, and large-cell OS.^8^ Histologically, OSs are composed of abnormal spindle cells that may produce osteoid or immature bone. In the jaw, the majority of the lesions reveal cartilaginous differentiation.^9^ Although OS of the jaw is better differentiated on histology than OS of the extremities, some tumors exhibit a deceptively bland histologic appearance, and hence, there is a need for appropriate clinicoradiopathologic correlation. Differentiation of OS and detection of any underlying bone lesion, such as odontogenic myomas, fibrous dysplasia, solitary plasmacytomas, Paget disease, and metastatic bone tumors, are based more on histopathology than radiologic evidence.^8,9^ Different levels of cell differentiation pose difficulty in distinguishing primary OS from reactive stromal proliferations that are commonly seen in callus formation in fractured bone and also in cellular forms of Paget disease.^3,9^ The patient reported here was diagnosed to have primary osteoblastic OS. Biochemical investigations that have been reported to have role in diagnosis are serum alkaline phosphatase and osteocalcin levels. Raised serum alkaline phosphatase may be seen in OS but is not a consistent finding. Osteocalcin, a bone-specific protein, has been used in differentiating OS from malignant fibrous histiocytoma.^7,9^

Apart from necrosis, various other potential prognostic markers that have been identified include tumor cell ploidy, expression of HER2/CerbB2, gains and loss of specific chromosomes, loss of *RB* and *p53* gene heterozygosity, and overexpression of P-glycoprotein.^3^ *MDM2* overexpression and amplification are found to be molecular makers for low-grade and dedifferentiated mandibular OS and are not seen in lesions such as fibrous dysplasia and ossifying fibroma. *GNAS* mutations that are specific for fibrous dysplasia are absent in low-grade OS. Juvenile ossifying fibromas have the potential to evolve into a giant-cell–rich variant of high-grade OS and are shown to be associated with *RASAL1* amplification or *RASAL1/MDM2* coamplification.^10^

Local recurrence and metastasis from jaw OS are less common than in OS of the long bones, and the proposed routes of metastatic spread include microscopically through the marrow spaces, via the mandibular canal or mental foramen and spread along the inferior alveolar nerve or mental nerve, and via the connective tissue that connects the intraosseous components and soft tissues such as the periodontal ligament. This may explain the facilitation of spread of an intraosseous lesion into the adjacent soft tissues. Metastases are relatively higher in postradiation OS of the jaws and tend to occur within 2 to 3 years. The 5-year survival rates of mandibular and maxillofacial OS are 34.8% and 25.8%, respectively, with a median survival time of 2.9 years for maxillary OS and 6.5 years for mandibular OS.^1,11,12^ Older age at onset carries a better prognosis because older age is associated with better resistance.^11,12^ The higher local recurrence rate (range, 33% to 39%) is attributed to the complex anatomy associated with the maxillofacial region that interferes with achieving adequate margins.^1^ In the patient presented here, the tumor showed an aggressive biologic course, an uncommon feature of gnathic lesions.

Unlike for extremity OS, for unknown reasons, chemotherapy has shown negligible impact on the outcome of craniofacial OS.^13^ Some studies have used radiotherapy and/or chemotherapy in conjunction with local radical surgery.^14^ Early diagnosis and appropriate surgery with adequate margins are the mainstay of treatment.^1,13,14^ In the patient presented here, no pulmonary or other distant metastases were present on whole-body scanning at presentation. Our patient’s disease was confirmed by histopathology and promptly treated by surgical resection. The patient was observed regularly at this center, and she responded well to the treatment; however, 8 months later, she developed skeletal and pulmonary metastases from the breast primary tumor.

In conclusion, gnathic OS is an uncommon mimic of common dental pathologies. Lack of awareness to consider OS in the maxillofacial region leads to delayed diagnosis that impedes early surgery. Timely radiopathologic diagnosis and depiction of extent of disease using three-dimensional volume-rendered CT and MRI are essential for prompt surgical resection and use of subsequent chemotherapy or radiotherapy.
